# Development of a clinical and translational research curriculum for undergraduate students

**DOI:** 10.1017/cts.2023.532

**Published:** 2023-05-02

**Authors:** Laura James, Tara Venable, Andres Caro, Jeffrey H. Moran, Claire Nesmith, Matthew A. Gannon, Lawrence E. Cornett

**Affiliations:** 1 The Departments of Pediatrics, University of Arkansas for Medical Sciences, Little Rock, AR, USA; 2 Department of Chemistry, Hendrix College, Conway, AR, USA; 3 Department of Pharmacology and Toxicology, University of Arkansas for Medical Sciences, Little Rock, AR, USA; 4 Community Health and Research, University of Arkansas for Medical Sciences, Little Rock, AR, USA; 5 Department of Physiology and Cell Biology, University of Arkansas for Medical Sciences, Little Rock, AR, USA

**Keywords:** Team science, undergraduate, translational research, cross-disciplinary research, career development, biomedical research

## Abstract

**Introduction:**

Research participation during undergraduate years has a powerful influence on career selection and attitudes toward scientific research. Most undergraduate research programs in academic health centers are oriented toward basic research or address a particular disease focus or research discipline. Undergraduate research programs that expose students to clinical and translational research may alter student perceptions about research and influence career selection.

**Methods:**

We developed an undergraduate summer research curriculum, anchored upon a clinical and translational research study developed to address a common unmet needs in neonatal nurseries (e.g., assessment of neonatal opioid withdrawal syndrome). Program topics reflected the cross-disciplinary expertise that contributed to the development of this “bedside to bench” study, including opioid addiction, vulnerable populations, research ethics, statistics, data collection and management, assay development, analytical laboratory analysis, and pharmacokinetics. The curriculum was delivered through three offerings over 12 months, using Zoom video-conferencing due to restrictions imposed by the COVID-19 pandemic.

**Results:**

Nine students participated in the program. Two-thirds reported the course enhanced their understanding of clinical and translational research. Over three-quarters reported the curriculum topics were very good or excellent. In open-ended questions, students reported that the cross-disciplinary nature of the curriculum was the strongest aspect of the program.

**Conclusion:**

The curriculum could be readily adapted by other Clinical and Translational Science Award programs seeking to provide clinical and translational research-oriented programs to undergraduate students. Application of cross-disciplinary research approaches to a specific clinical and translational research question provides students with relevant examples of translational research and translational science.

## Introduction

Participation in research and mentorship during undergraduate years is known to have a powerful influence on career selection in scientific and engineering fields [1–4]. Undergraduate research opportunities have traditionally been linked to basic science laboratories [5,6]. While summer undergraduate biomedical research programs exist in many academic health centers across the USA, many of these are laboratory based, highlight a specific disease focus or research discipline and are not necessarily affiliated with Clinical and Translational Science Awards at their respective institutions [7]. The extent to which existing undergraduate research opportunities emphasize the principles of team science, translational research, and translational science [8–10], is unknown.

In the following manuscript, we report our experience developing and administering training in clinical and translational research for undergraduate students. Our curriculum aligns with recent definitions of team science, whereby the “strengths and expertise of professionals trained in different fields work together to address scientific challenges” [11]. The novelty of the curriculum is that it was anchored upon a clinical and translational research study which received professional contributions from numerous cross-disciplinary researchers in its development, design, and conduct. The curriculum could be adapted by other academic medical centers and Clinical and Translational Science Awards (CTSAs) seeking to educate undergraduate students in the principles of clinical and translational research.

Support for development of the curriculum was provided through a supplement (NIH P20 GM103429-19S1) to the parent grant that supports the Arkansas IDeA Network of Biomedical Research Excellence (INBRE). The Institutional Development Awards (IDeA) program was established by Congress in 1993 to expand the geographic distribution of National Institutes of Health (NIH) funding to states with historically low rates of federal research funding. The IDeA Networks of Biomedical Research Excellence (INBRE) award program fosters the development, coordination, and sharing of research resources and expertise to expand research opportunities and increase the number of competitive investigators in IDeA-eligible states. The Arkansas INBRE promotes biomedical research with programs for undergraduate students and faculty statewide. The University of Arkansas for Medical Sciences (UAMS) and the University of Arkansas serve as the lead institutions for the Arkansas INBRE, which has impacted nearly all colleges and universities in the state during its 21-year history.

The Arkansas INBRE offers a mentored summer research program that provides opportunities for undergraduate students a research experience with established biomedical researchers at the lead institutions. Nearly all host research laboratories focus on basic science approaches and/or early-stage translational or T1 research, traditionally defined as the development and validation of animal models and/or preclinical drug studies. The UAMS CTSA, known as the Translational Research Institute, or TRI, provides resources to expand investigator training across the T1–T4 translational research spectrum. A supplement award to the UAMS INBRE (NIH grant P20 GM103429) provided the opportunity for TRI and the Arkansas INBRE to develop a curriculum for undergraduate students framed upon a clinical and translational research (CTR) study.

## Materials and Methods

A representative CTR study supported by institutional funds to develop opioid-related research and INBRE funding provided a framework for creating a unique curriculum for undergraduate students that exposed the students to numerous principles of CTR. Opioid addiction is a common and persistent challenge that has generational effects on the US population [12]. The CTR study involved cross-disciplinary collaborations from numerous investigators working across multiple departments and provided a compelling example of “bedside to bench side” translational research, illustrating the bidirectional nature of translational research. The CTR study was developed to provide new information in response to an unmet need identified by practicing clinicians [12,13] in the neonatal nursery (described below). In addition, participating students were exposed to the principles of team science. This broader exposure allows students to learn how experts from multiple disciplines work collaboratively on a single project to develop and test a translational research question.

### Clinical Background and Gap in Patient Care Management

The management of opioid dependency in pregnancy has dramatically shifted over the last two decades [14]. The majority of medical centers now use buprenorphine rather than methadone as maintenance opioid treatment during pregnancy [15]. This change in practice stems from reports demonstrating the efficacy and safety of buprenorphine, compared to methadone, for the management of opioid dependency in pregnancy. Infants of buprenorphine-treated mothers have less cardiac suppression and reduced lengths of hospitalization, compared to infants of methadone-treated mothers [16]. Unfortunately, the management of infants at risk for developing neonatal opioid withdrawal syndrome (NOWS) lacks consistency across institutions [17] and many hospitals require a minimum of 72 hours of mandatory monitoring prior to hospital discharge. This practice is expensive and lacks an evidence base.

Clinical assessment for NOWS relies on the Finnegan scale [18] which was developed initially to characterize infant withdrawal through assessment of neurodevelopmental markers (e.g., feeding, cry, tone, stooling pattern, temperature regulation, and irritability). The modified Finnegan scale is widely used today and guides both nonpharmacological and pharmacological interventions to help alleviate NOWS [19]. Other than the Finnegan scale, there are no standard approaches to diagnose, predict, or quantify the likelihood that a neonate will or will not show signs of withdrawal [20].

### Development of the Clinical and Translational Research (CTR) Curriculum for Undergraduates

The primary goal of the CTR study was to quantify buprenorphine and its primary metabolite nor-buprenorphine in infants born to mothers receiving buprenorphine for the treatment of opioid dependency in pregnancy. Buprenorphine and norbuprenorphine levels were compared to established clinical scoring for NOWS. The research protocol focused on defining the relationship between measurements of buprenorphine and norbuprenorphine in infant blood samples to the development of NOWS. Prior to the initiation of the clinical research protocol, a new assay for the measurement of buprenorphine and nor-buprenorphine in infant whole blood samples was developed and validated in a CLIA-accredited laboratory. Whole blood samples were extracted using standard supported liquid extraction techniques and analyzed using liquid chromatography tandem mass spectrometry (LC-MS/MS).

The clinical research protocol was developed by a clinical pharmacologist with extensive input from a team of pediatric hospitalists working in the neonatal nursery and neonatal intensive care unit. The lead pediatric hospitalists obtained additional consultation from the clinical community, including obstetricians, clinical nursing staff, and pregnant mothers with opioid addition who participated in a support group led by the institution’s Department of Psychiatry. The protocol received approval from the UAMS Institutional Review Board and informed consent from mothers was required. The blood sampling strategy for the clinical research protocol was tailored to accommodate the small circulating blood volume of infants by obtaining samples at times where blood sampling was scheduled for routine clinical monitoring of the infants. Umbilical cord blood samples were collected from infants at the time of delivery and from infants at 24 and 72 hours after birth. Modified Finnegan scores were captured from the Epic electronic health record and downloaded into a study specific REDCap database.

### Curriculum Offering and Undergraduate Student Selection

Participating students were recruited from Hendrix College (Conway, Arkansas). The original program was designed to be a 12-week on-site summer program during which students would rotate in various laboratories and the neonatal nursery. When the COVID-19 pandemic precluded in person meetings, the program was modified to use a Zoom video-conferencing format and was offered during three semesters over a 12-month period. Lecture topics for the curriculum (Table 1) illustrate the cross-disciplinary nature of the curriculum and provide an example of engaging all relevant expertise across disciplines, fields, and professions to produce research that advances translation [12].

**Table 1.** Curriculum for clinical and translational research immersion for undergraduate students

Introduction to clinical and translational research Virtual tour of neonatal nursery Global perspectives on opioid addiction Drug metabolism Mass Spectroscopy Research data bases Pharmacokinetics and statistics Scientific presentations

### Overview of CTR Curriculum

Titles of lectures are listed in Table 1. Each session used Zoom video-conferencing and was attended by all students, the undergraduate advisory faculty member, and the assigned faculty lecturer. The first lecture provided a course overview, a review of clinical and translational research, a brief history of research ethics, and introductory information about neonatal nurseries. Students participated in a “remote tour” of the nursery led by the neonatal nursery faculty. Using a computer-on-wheels, the faculty video-conferenced with the students in real-time and provided them with an orientation of a neonatal intensive care room, including the neonatal incubator bed, warmer, and monitoring equipment. The faculty addressed various nursery topics, including treatment approaches commonly used for infants with NOWS. The students were also shown a neonatal resuscitation room in the Labor and Delivery unit, further re-iterating the importance of team-based clinical care and research. Additional topics provided in the curriculum included foundational exposure to human subjects research, regulations around research in vulnerable populations, maternal and neonatal clinical care, opioid addiction and treatment, knowledge gaps in clinical care, cross-disciplinary research team building, drug metabolism, laboratory testing and quantitative analysis, pharmacokinetic analysis, clinical scoring systems, clinical pharmacology, clinical informatics, data management, statistical analysis, and scientific presentations. Each lecture was approximately 90 minutes in nature and question and answer opportunities were included in each session.

### Evaluation

Students completed a survey about the curriculum following course completion. The survey included Likert-scale questions about the students’ overall experience of the course, with response options of strongly agree, agree, neutral, disagree, and strongly disagree. A second set of questions on their satisfaction with the individual modules had response options of excellent, very good, good, fair, and poor.

## Results

Nine students (three males; six females) participated in the program. The median (and range) student age were 21 (19–23) years. Seven of the students identified as White, one identified as Asian, and one identified as both White and Hispanic. The academic year for the participating students was one sophomore, five juniors, and three seniors. After graduation from Hendrix College, all students were planning on attending either medical school or graduate school.

**Figure 1.**
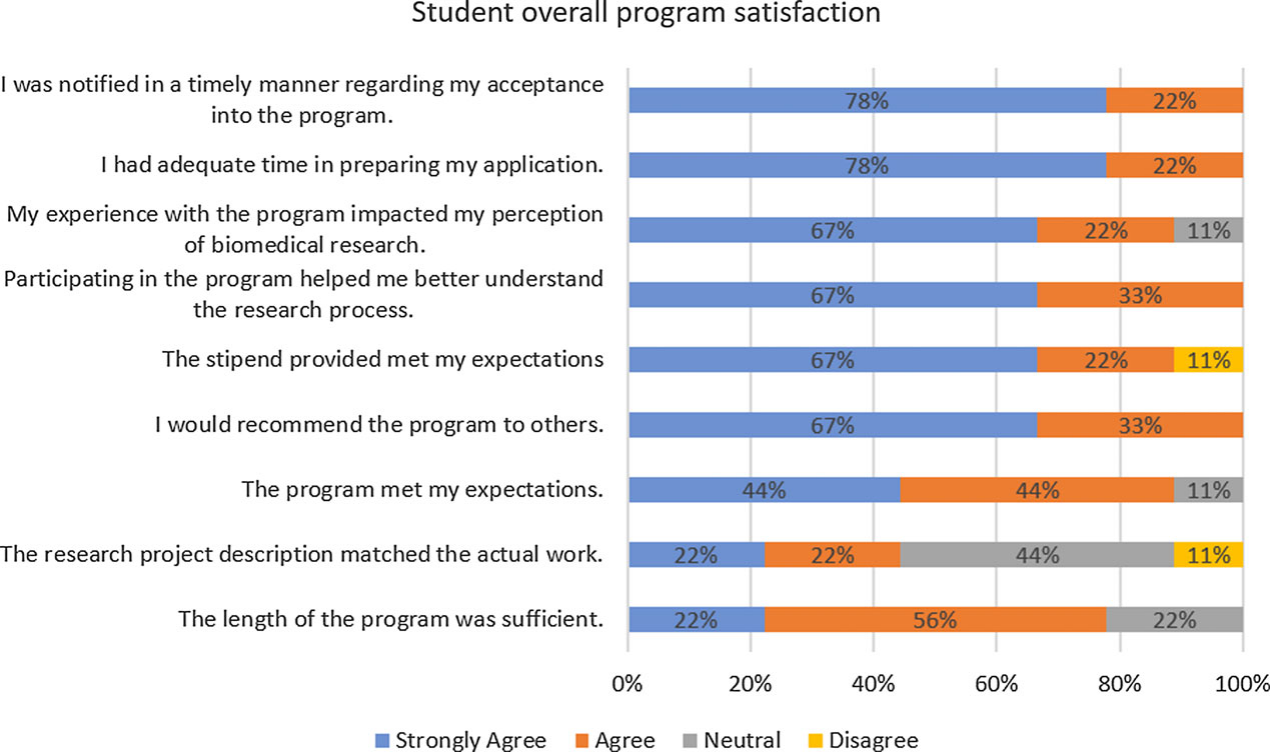
Program satisfaction among participating students.

**Figure 2.**
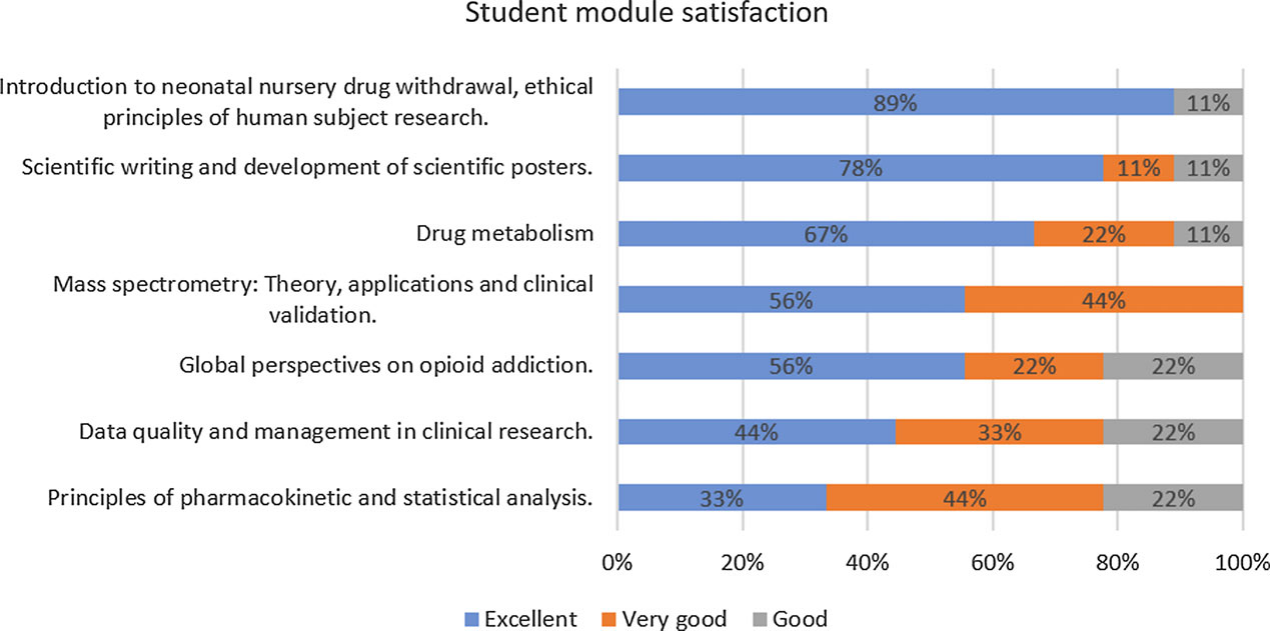
Module satisfaction among participating students.

Overall, the students were very favorable about their experience, with over two-thirds of participants stating they agreed or strongly agreed that the course met their expectations, they would recommend the course to others, and felt that the course enhanced their understanding of research. Satisfaction was also high for the individual program modules, with all modules being rated as excellent or very good by more than three-quarters of the students. Response frequencies to all the questions asked are shown in Figs. 1 and 2. In open-response questions, students indicated that the cross-disciplinary nature of the program was among the most important aspects and several mentioned that their career goals had changed to include more emphasis on biomedical research.

## Discussion

The developed CTR curriculum provided new educational materials that were administered by clinical and research faculty who were well versed in the topic areas through their training, experience, and contributions to development of the research study. These faculty participated in either the development (study design, data management, assay development) and/or clinical implementation (neonatal nursery clinicians) of the study. The “real world experience” of the CTR study provided a cohesive framework for the curriculum whereby clinical and translational research concepts were illustrated through application of the concepts to a specific research question. Our curriculum could be adapted by other CTSA programs, whereby one (or more) representative clinical and translational research studies are selected as case studies and cross-disciplinary experts involved in study development demonstrate their discipline-specific contributions to the study. Many of the lecture topics reflect core aspects of CTR that are well developed in CTSA programs (e.g., research ethics, study design, statistical analysis, and research data management). Additional curriculum topics for inclusion could be a) an introduction to the clinical context from which the research question was developed, b) exposure to additional scientific content expertise, and c) presentations of research ethics relevant to the representative studies.

In open-ended questions provided to the students, they indicated that the strongest component of the curriculum was the cross-disciplinary nature of the program. The curriculum appeared to provide a compelling example to students of how CTR concepts can be applied to solve an unmet need in the clinical care of a vulnerable population [12]. This example successfully helped students understand CTR to the extent that 89% noted that the experience altered their perceptions of research and 100% of the students noted that the experience helped them better understand research conducted in academic health centers.

While our evaluation of the curriculum did not include a learning assessment, the data are particularly noteworthy given that all meetings between students and faculty were conducted virtually as a result of the COVID-19 pandemic. Other groups have demonstrated high levels of satisfaction with summer research programs that were transformed into a virtual format as a result of the COVID-19 pandemic [21,22].

Finally, summer research programs for undergraduate students have been shown to impact their pursuit of additional training and ultimately their career choices [23]. The recent publication by Faupel-Badger [12] emphasized the need to expose undergraduate students to translational research and translational science early in their education before critical decision-making time periods of professional development. Our early experience with a CTR curriculum for undergraduate students suggests that student perceptions of biomedical research can be influenced, even through a virtual exposure experience. We plan long-term follow-up with these students and additional studies to assess whether research experiences that are translational in nature steer students to pursue careers that impact human health through research.
